# Nicotine or tobacco abstinence?

**DOI:** 10.1183/16000617.0089-2022

**Published:** 2022-11-02

**Authors:** Rachael L. Murray, Matthew Evison, Matthew E. Callister

**Affiliations:** 1 Academic Unit of Lifespan and Population Health, School of Medicine, University of Nottingham, Nottingham, UK; 2 SPECTRUM consortium, Edinburgh, UK; 3 Wythenshaw Hospital, Manchester University NHS Foundation Trust, Manchester, UK; 4 Leeds Teaching Hospitals NHS Trust, Leeds, UK

## Abstract

We read with interest the meta-analysis by Hanewinkel *et al.* [1] regarding e-cigarette use and nicotine abstinence. The summary graph from the manuscript (shown here as figure 1a) clearly illustrates the competing issues in comparing the harms or benefits of these two smoking cessation adjuncts.

## Abstract 2

**Tobacco abstinence should be the key public health goal to reduce the mortality and morbidity burden in people who smoke; nicotine is not the key substance responsible for the incidence of many cancers, heart and respiratory disease from smoking** https://bit.ly/3QdrqR1

*To the Editor:*

We read with interest the meta-analysis by Hanewinkel *et al.* [1] regarding e-cigarette use and nicotine abstinence. The summary graph from the manuscript (shown here as figure 1a) clearly illustrates the competing issues in comparing the harms or benefits of these two smoking cessation adjuncts.

**FIGURE 1:**
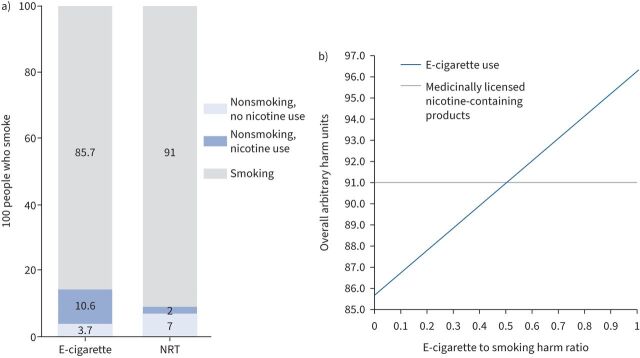
a) Anticipated absolute effects of e-cigarette use *versus* nicotine replacement therapy (NRT); reproduced from Hanewinkel *et al.* [1]. b) Overall net population harm caused by the use of e-cigarettes *versus* medicinally licensed nicotine-containing products according to e-cigarette to smoking harm ratio.

Considering the population as a whole, 85.7% of people will continue to smoke and 3.7% will quit nicotine altogether irrespective of the smoking cessation adjunct used. This leaves 10.6% of the population who are of interest in this comparison. In the e-cigarette group, all 10.6% continue to use nicotine through their e-cigarette. In the nicotine replacement therapy (NRT) group, 5.3% continue to smoke, 2% continue to use nicotine through NRT, and 3.3% quit nicotine use altogether.

The public health goal is to effectively treat tobacco dependence, and thus reduce smoking prevalence. Whilst not completely harmless, medicinally licensed nicotine does not evoke the same health risks and harms (*e.g.* cardiovascular disease, cancer, death) that occur from smoking tobacco. It is for this reason the National Institute for Health and Care Excellence (NICE) in the UK recommend medicinally licensed nicotine-containing products are prescribed for as long as is required to prevent relapse to smoking without any time limit [2].

In comparing the overall harms between these two approaches, we are therefore attempting to balance the harms to the 5.3% of participants in the NRT group who continue to smoke against the 10.6% of participants in the e-cigarette group who continue long-term use of nicotine through their e-cigarette. The obvious uncertainty here is the ratio of harm between e-cigarette use and smoking. Although there is huge divergence in opinion on e-cigarettes, areas of probable consensus are that e-cigarettes are unlikely to be completely harmless, and that e-cigarettes are unlikely to be more harmful than smoking. It is likely, therefore, that the ratio of e-cigarette harm relative to smoking will be somewhere between 0 and 1.

This precise harm ratio will only become apparent with the passage of time. However, simple mathematical modelling can inform the debate in a non-partisan way in the interim. If we define an arbitrary harm unit (AHU) as the annual harm done by smoking cigarettes, then it is straightforward to model the harm that might be done by vaping as the harm ratio varies as shown in figure 1b. For the 100 people in the NRT group, the overall harm will be 91 AHUs (the number who continue to smoke with NRT). The harm in the e-cigarette group will vary from 85.7 AHUs (if e-cigarettes are harmless) to 96.3 AHUs (if e-cigarettes are as harmful as normal cigarettes). Therefore, if the harm of e-cigarettes is >50% that of smoking, then their use results in net harm to the population as a whole, whereas if the harm ratio is <50%, there is a net benefit to population health. Public Health England has previously estimated the harm of e-cigarette use to be 5% that of smoking tobacco [3], although this estimate has been disputed [4]. At this harm ratio, the overall harm of medicinally licensed nicotine-containing products in this population would be 91 AHUs *versus* 86.2 AHUs for the vaping population.

In summary, if e-cigarette use does less than half the harm of smoking tobacco, their use would appear to result in a net gain in population health in comparison to medicinally licensed nicotine-containing products when treating patients with tobacco dependency. Whilst e-liquid aerosols contain harmful chemicals, the NICE tobacco dependency guideline group in the UK concluded vaping is likely to be “substantially less harmful than smoking tobacco” following a network meta-analysis by the expert guideline committee using the standard robust NICE methodology [2]. We propose that the data described by Hanewinkel *et al.* [1] have provided further evidence that supports the use of e-cigarettes as an evidence-based intervention for tobacco dependency when the primary goal is tobacco abstinence. However, as stated above, e-cigarettes are unlikely to be completely harmless, and thus alongside their use as an evidence-based treatment for tobacco dependency, it is vital that their use in never-smokers, particularly teenagers, is heavily discouraged due to uncertainty about long-term effects.
